# Context-dependent regulation of IL-6 in exercise and colorectal cancer cachexia

**DOI:** 10.3389/fonc.2026.1792184

**Published:** 2026-07-10

**Authors:** Jingwen Xiong, Sihan Liu, Jie Xu, Jinghan Zhang, Yundong Peng, Yibei Xia, Jinghua Qian

**Affiliations:** 1 School of Sports Medicine and Rehabilitation, Beijing Sport University, Beijing, China; 2 Key Laboratory of Exercise Rehabilitation Science of the Ministry of Education, Beijing Sport University, Beijing, China; 3 School of Cardiovascular & Metabolic Health, College of Medicine, Veterinary & Life Sciences, University of Glasgow, Glasgow, United Kingdom; 4 West China School of Medicine, West China Hospital, Sichuan University, Chengdu, China; 5 Department of Anesthesiology, People’s Hospital, Peking University, Beijing, China

**Keywords:** cachexia, colorectal cancer (CRC), exercise oncology, exercise therapy, IL-6

## Abstract

Colorectal cancer (CRC) is a common malignancy of the digestive system and remains a major cause of cancer-related morbidity and mortality worldwide. Its development and progression are influenced by genetic susceptibility, lifestyle factors, metabolic dysregulation, and chronic inflammatory states. Cancer cachexia is a multifactorial wasting syndrome characterized by weight loss, skeletal muscle atrophy, metabolic disturbances, and functional decline, which substantially impairs quality of life and survival outcomes in patients with CRC. In this context, interleukin-6 (IL-6) has emerged as a context-dependent regulatory mediator linking inflammation, metabolism, tumor biology, and skeletal muscle wasting. The biological effects of IL-6 are shaped by signaling mode, cellular source, concentration, temporal dynamics, and the broader tumor–host environment. This review discusses the regulatory roles of IL-6 in CRC and CRC-related cachexia, with particular attention to exercise-associated IL-6 responses. Rather than viewing exercise-induced IL-6 as inherently beneficial or antitumor, we emphasize that IL-6 is one component of a broader exercise-responsive network involving metabolic adaptation, immune regulation, skeletal muscle function, and systemic inflammatory remodeling. We also summarize current evidence regarding how exercise modality and intensity may influence IL-6 dynamics, while distinguishing mechanistic and preclinical findings from limited and heterogeneous human data. Overall, IL-6 dynamics may provide a hypothesis-generating framework for understanding exercise responses in CRC-related cachexia, but current evidence is insufficient to support IL-6-guided individualized exercise prescription or therapeutic decision-making in clinical practice.

## 1 Introduction

CRC is the third most common cancer worldwide, accounting for approximately 10% of all cancer cases and deaths (1). The incidence of CRC is positively correlated with the Human Development Index (HDI) (2). A chronic inflammatory environment caused by infections, abnormal immune responses or environmental factors is strongly associated with tumor development (3). CRC development is largely associated with a chronic inflammatory environment, with long-standing, poorly controlled inflammatory gastrointestinal disease being a major risk factor (4). A chronic inflammatory environment may induce insulin resistance and hyperinsulinemia, increasing insulin and Insulin-like growth factor-1 (IGF-1) levels that promote colon cell proliferation, potentially contributing to CRC development (5). This persistent chronic inflammatory state is not only an increased risk of tumorigenesis, but also a key pathological basis that drives cancer cachexia (6). Cancer-associated cachexia is a multifactorial syndrome characterized by loss of skeletal muscle and adipose tissue, systemic inflammation, and weight loss, leading to reduced muscle strength, impaired physical function, and decreased chemotherapy tolerance (7). Cachexia has the highest incidence in lung cancer and CRC (8), and the prevalence rate in CRC is as high as 60%, becoming the direct cause of death in at least 20% of patients (9).

IL-6 belongs to the IL-6 cytokine family, which also includes IL-11, IL-27, IL-35, IL-39, leukemia inhibitory factor (LIF), oncostatin M (OSM), cardiotrophin-1 (CT-1), CT-2, cardiotrophin-like cytokine (CLC), and ciliary neurotrophic factor (CNTF) (10). IL-6 has been identified as an important “bridge” between chronic inflammation and malignant tissue (11). The plasma concentration of circulating IL-6 is associated with the progression of cachexia in advanced cancer patients (12). Cachexia is often accompanied by insulin resistance, decreased muscle mass, and the development of chronic inflammation. Sarcopenia and neutrophil to lymphocyte ratio (NLR) are emerging as new prognostic markers for CRC (13). To date, circulating IL-6 is widely recognized in inflammatory diseases, obesity-related diseases and cancer. However, IL-6 has context-dependent and sometimes opposing biological effects. In 2000, Steensberg et al. reported for the first time that IL-6 produced by skeletal muscle contraction entered the circulation (14). This significant finding attracted considerable research attention. IL-6 contributes to the maintenance of glucose homeostasis. This finding contrasts with the conventional view that circulating IL-6 promotes insulin resistance. This study suggested that exercise-induced IL-6 contributes to the maintenance of glucose homeostasis. Both type I and type II muscle fibers express IL-6 and produce effects locally in the muscle. Once IL-6 is released into the blood circulation, it demonstrates its function as a hormone in several organs (15). In addition, IL-6 also enters the circulation in the form of exosomes (16), but this is not the focus of our discussion.

Exercise has been reported to have an ameliorative effect on 13 types of cancer (17). Studies have found that appropriate frequency of physical activity can mitigate cachexia (18). Although the specific mechanism by which exercise improves CRC cachexia has not been elucidated, it may include exercise-induced changes in growth factors and inflammatory factors (19). Various physiological and metabolic changes caused by exercise can cause a range of positive effects, including increased insulin sensitivity, reduced inflammatory responses and improved muscle mass. IL-6 is usually the first myokine detected in circulation and it is currently the most studied myokine. It exhibits one of the most pronounced exercise-induced changes among myokines (20). By being released into circulation, IL-6 strengthens the link between exercise and cancer. In the tumor microenvironment (TME), tumor pro-inflammatory factors and immune cells are active, and exercise-induced IL-6 interacts extensively with these inflammatory and immune components.

Circulating IL-6 plays a complex role in CRC pathology, and exercise, as a non-pharmacological intervention, modulates IL-6 and other related biomarkers, thereby mitigating cachexia, inhibiting tumor growth, and improving patient survival outcomes.

## 2 Overview of cancer cachexia

During inflammation, tumor cells release inflammatory factors, including tumor necrosis factor, IL-6, and IL-8, which promote skeletal muscle atrophy by enhancing protein degradation and inducing oxidative stress (21). Increased level of circulating IL-6 and tumor necrosis factor-α (TNF-α) may lead to muscle inflammation. They induce insulin resistance by activating the Nuclear Factor kappa-light-chain-enhancer of activated B cells (NF-κB) and other signaling pathways, which promote protein degradation and impair insulin signaling (22). In addition, circulating IL-6-induced insulin resistance may also be due to the upregulation of suppressor of cytokine signaling 3 (SOCS3), which inhibits the expression of the insulin receptor (23). Elevated inflammatory cytokines also activate the ubiquitin–proteasome pathway, leading to insulin resistance and muscle atrophy, thereby exacerbating the patient’s sarcopenia (24). In addition, elevated levels of reactive oxygen species (ROS) can induce oxidative stress, leading to mitochondrial dysfunction and muscle wasting (25). A decrease in muscle mass may also impair glucose metabolism, thereby contributing to insulin resistance (26), forming a vicious circle.

Sarcopenia may be more pronounced in cancer patients who are influenced by obesity factors. Pro-inflammatory factors can induce immune cells to infiltrate into fat and muscle, thereby releasing more pro-inflammatory factors, causing fat depots and localized muscle inflammation to progress to low-grade systemic inflammation, exacerbating insulin resistance and lipid dysfunction (27). In addition, leptin secreted by adipocytes can act on monocytes to induce the release of TNF-α and IL-6 (28). However, IL-6 cannot regulate leptin levels alone (29). TNF-α and IL-6-induced disturbances in muscle metabolism create a self-reinforcing vicious cycle that not only exacerbates localized muscle loss but also promotes a systemic inflammatory state that exacerbates the severity of sarcopenia. Such a cycle of inflammation may not only enhance the aggressiveness of the tumor but may also diminish the response to treatment.

## 3 Molecular basis of IL-6

**Figure 1:**
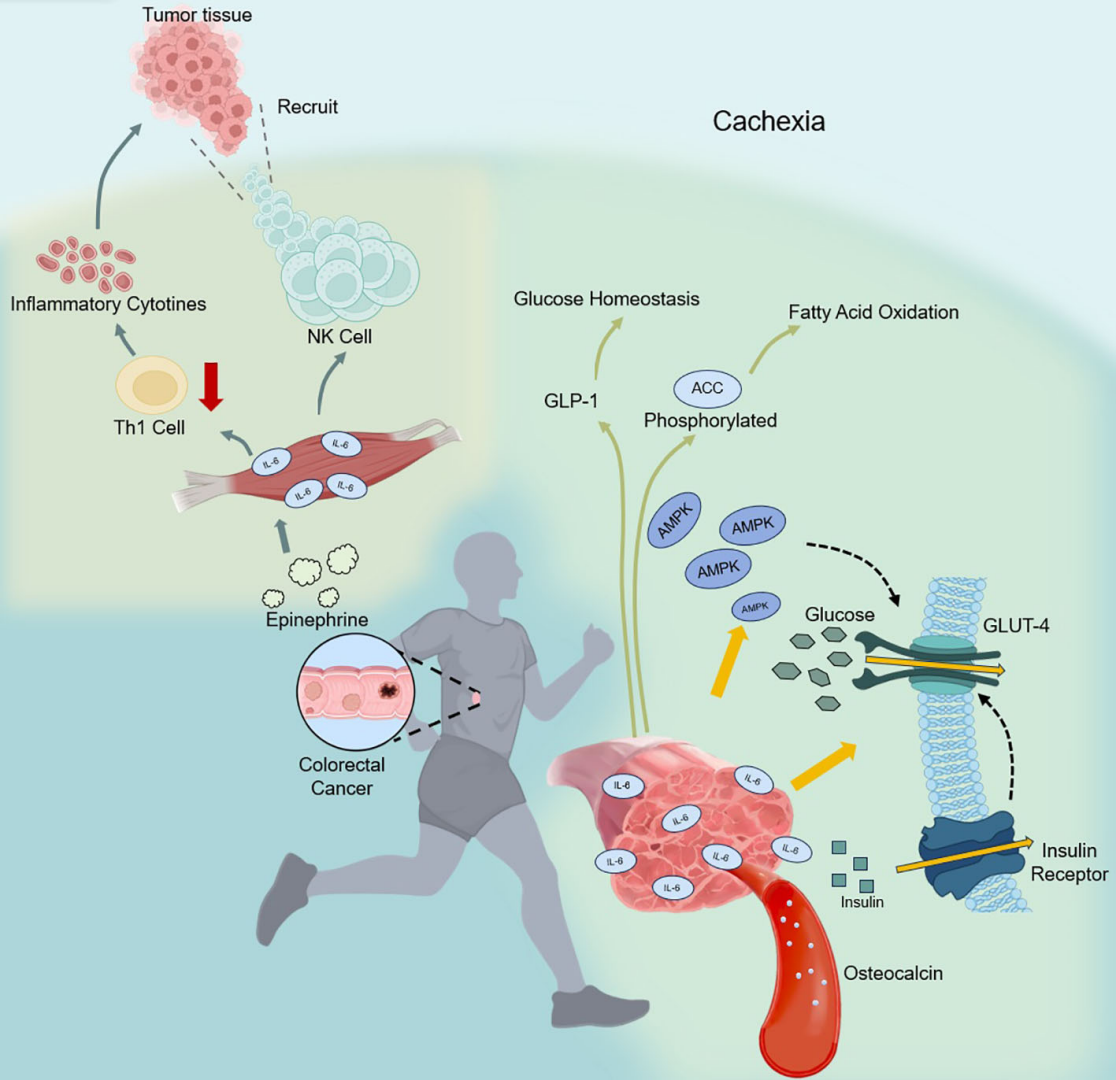
In the context of colorectal cancer cachexia, exercise stimulates skeletal muscle to release IL-6, which promotes GLP-1 synthesis and secretion by acting directly or indirectly on intestinal L cells and pancreatic alpha cells, thereby improving glucose homeostasis. IL-6 activates AMPK, on the one hand, induces GLUT-4 to migrate to the cell membrane to enhance glucose uptake; on the other hand, it inhibits its activity by phosphorylation ACC, and finally promotes fatty acid oxidation. In addition, exercise can also increase muscle sensitivity to insulin, making insulin more effective at lower concentrations to promote glucose uptake and utilization. IL-6 can also further enhance the metabolic adaptation of the body to exercise by increasing the level of bioactive osteocalcin. IL-6 induced by exercise can promote NK cell infiltration into tumor microenvironment, but inhibit Th1 type immune response. This mechanism was more significant in the presence of epinephrine, suggesting that IL-6 has a complex bi-directional regulatory role in tumor immunity.

IL-6 is a key cytokine linking inflammatory responses, energy metabolism, immune regulation, and skeletal muscle homeostasis; however, its concentration dynamics and biological significance vary markedly across physiological and pathological conditions (**Figure 1**).

### 3.1 IL-6 signaling pathways

IL-6 acts through a variety of different pathways, including classical signaling, trans signaling, cluster signaling, and intracellular autocrine signaling. Classical signaling occurs when IL-6 binds to the membrane bound IL-6 receptor (IL-6R), which recruits universally expressed glycoprotein 130(gp130) to initiate an intracellular signaling cascade; this pathway is primarily anti-inflammatory or regenerative and supports acute phase responses of hepatocytes, plasma cell differentiation, and tissue reparation (30, 31). In contrast, trans signaling occurs when IL-6 binds to the soluble IL-6 receptor (sIL-6R), activating signals via gp130 on cells that do not express membrane IL-6R, thereby expanding the range of responding cells; this pattern is primarily proinflammatory, driving monocyte recruitment, monocyte chemoattractant protein-1 (MCP-1) secretion by endothelial cells, and T helper 17 (Th17) cell maintenance (31, 32). The balance between classical and trans signaling is affected by IL-6R expression levels, sIL-6R production by ADAM17/ADAM10 protease cleavage, and the presence of soluble gp130 (33, 34). Membrane IL-6R is mainly expressed on hepatocytes, naive/central memory T cells and some leukocytes, while activated effector T cells tend to downregulate IL-6R so that it responds mainly by trans signaling. In addition, cluster signaling (35) occurs in membrane-bound IL-6, IL-6R complex located on the sender cell and activates gp130 in adjacent recipient cells, a process that can be inhibited by extracellular soluble gp130 (sgp130-Fc) and plays an important role in T cell transduction and IL-11 signaling. It is worth noting that IL-6 and IL-11 can also act through intracellular autocrine mechanisms, with the gp130 complex activating signal transducer and activator of transcription 3 (STAT3) signaling inside the cell, initiating the signal before it is secreted.

Some studies have identified IL-6 splice variants that may exhibit cell type-specific biological activities (36–38). However, their functional significance remains unconfirmed, and the prevailing view still considers IL-6 as a single cytokine, with differential biological behavior mainly arising from signaling context and receptor environment rather than distinct protein isoforms.

Generally, classical signaling is anti-inflammatory and regenerative, whereas trans signaling is pro-inflammatory, cluster signaling mediates paracrine transduction, and intracellular autocrine signaling allows cells to self-activate without extracellular inhibition, with different functions depending on receptor localization, receptor cleavage, and cellular environment.

### 3.2 Context-dependent IL-6

#### 3.2.1 IL-6 in acute inflammation

During acute inflammation, IL-6 is rapidly induced by multiple cell types within the inflammatory milieu. In addition to classical sources like macrophages, fibroblasts, and endothelial cells, dendritic cells, monocytes, and barrier epithelial cells can also rapidly upregulate it in response to pathogen- or damage-associated molecular patterns (PAMPs or DAMPs). This induction is mainly mediated by innate immune recognition pathways and is often enhanced by pro-inflammatory cytokines such as TNF-α and IL-1β. Downstream, these signals converge on NF-κB- and MAPK-centered transcriptional programs, promoting IL-6 gene expression and thereby initiating classical the IL-6 signaling (39, 40). Concurrently, IL-6 acts on endothelial and smooth muscle cells to induce adhesion molecules and chemokine networks, driving production of IL-8 and MCP-1 (CCL2), leukocyte recruitment, acute-phase responses, and amplification of local inflammation (41, 42).

#### 3.2.2 IL-6 in chronic inflammation

In chronic inflammation, IL-6 trans-signaling in stromal cells shifts leukocyte recruitment from early neutrophils to sustained monocyte influx. IL-8-mediated neutrophil activation can promote shedding of membrane-bound receptors, thereby initiating IL-6 trans-signaling (43, 44). Recruited monocytes subsequently differentiate into macrophages and release pro-inflammatory cytokines, including IL-1β and TNF-α, thereby prolonging the inflammatory response. This effect can be further reinforced by the phagocytosis of apoptotic neutrophils (45). Together, IL-6 trans-signaling coordinates chemokine dynamics and immune cell interactions, maintaining chronic inflammation and tissue pathology.

#### 3.2.3 Exercise-induced IL-6

At rest, approximately 30% of circulating IL-6 originates from adipose tissue, primarily produced by resident macrophages rather than adipocytes (46), whereas skeletal muscle contains only low basal levels of IL-6, concentrated in type I fibers (47). During exercise, IL-6 primarily contributes to metabolic (48) and immune responses (49), as discussed in detail in the following sections.

#### 3.2.4 IL-6 associated with colorectal cancer

In mouse models of colitis associated CRC, approximately half of IL-6 in tumors is derived from hematopoietic cells, and epithelial cells produce a small fraction of IL-6. More importantly, IL-6 and its associated factors play an integral role in driving cancer development (50). IL-6 is often a key proinflammatory mediator in gut-associated tumors, and IL-8 is frequently overexpressed in CRC (51) and is associated with enhanced angiogenesis and increased metastatic potential. Inflammatory signaling networks driven by IL-6/IL-8 may mechanistically reinforce the link between oncogene-induced cell senescence and its inflammatory phenotype (SASP) and tumor progression (52). In colon cancer, IL-6 produced by stromal fibroblasts promotes enhanced fibroblast production of vascular endothelial growth factor (VEGF), thereby inducing tumor angiogenesis (53). IL-6 directly contributes to the development and progression of inflammation-associated CRC through multiple mechanisms. In Colitis-Associated Cancer (CAC), lamina propria macrophages promote aberrant epithelial proliferation and carcinogenesis through IL-6 trans-signaling (54). In parallel, CD90^+^ myofibroblasts and macrophages within the tumor stroma can continuously produce IL-6, further inducing Th17 inflammatory responses, characterized by IL-17A-producing helper T cells, and amplifying tumor-associated inflammation (55, 56). In addition, IL-6 trans-signaling can induce ROS generation, which specifically drives the mislocalization of the mismatch repair protein hMSH3 from the nucleus to the cytoplasm, leading to frameshift mutations in DNA tetranucleotide repeats and ultimately resulting in elevated microsatellite alterations at selected tetranucleotide repeats (EMAST) formation (57). Anti-IL-6 antibodies, sgp130 Fc, or STAT3 inhibitors have been shown to block this process, providing potential targets for intervention in inflammation-associated CRC (57).

Although IL-6 is commonly associated with chronic inflammation, tumor progression, immune suppression, and cachexia in colorectal cancer, its role in the tumor microenvironment should not be interpreted as uniformly tumor-promoting. Limited evidence suggests that, under specific tumor subtypes and immune contexts, IL-6 may also be involved in regulatory processes that shape antitumor responses. For example, in certain CRC clinical cohorts, higher IL-6 expression has been associated with improved prognosis (58), suggesting that the prognostic significance of IL-6 may be influenced by tumor subtype and immune infiltration status. Early experimental evidence also showed that, in selected colorectal tumor models, recombinant IL-6 significantly reduced established lung and liver metastases in mice bearing MC38 colon adenocarcinoma, although this effect was not observed across all tumor types (59). In addition, IL-6 may act as part of a broader cytokine network involved in the priming and activation of CD4^+^ and CD8^+^ T cells during antitumor immune surveillance (60). Notably, in the CT26 colon cancer model, IL-6 blockade reshaped checkpoint blockade-induced immune responses by reducing Th17-skewed inflammation while increasing intratumoral Th1 and CD8^+^ effector T-cell density, indicating that IL-6 signaling can influence the balance between inflammatory toxicity and effective antitumor immunity (61). Therefore, the role of IL-6 in CRC should be viewed as context-dependent immune regulation shaped by tumor type, immune context, signaling mode, and temporal dynamics, rather than as a fixed tumor-promoting or antitumor effect.

## 4 Exercise-induced IL-6 levels: influence of exercise parameters

IL-6 concentrations are influenced by a variety of exercise parameters (exercise duration, intensity, type and frequency). Exercise duration appears to be the most important determinant of circulating IL-6 concentration, followed by intensity of exercise (62). Circulating IL-6 is produced by skeletal muscle contraction after exercise. And its level can increase rapidly (63). After exercise, circulating IL-6 levels can increase rapidly (64). However, the exercise-induced increase in IL-6 concentration is not linear but follows an exponential pattern (62). Studies have indicated that the half-life of exercise-induced IL-6 is approximately 5 minutes (65). The effective half-life of IL-6 in plasma is modulated by soluble factors such as sIL-6R and sgp130 and may be prolonged under pathological conditions (66), manifesting as sustained elevations in patients with inflammatory disorders (67). IL-6 mRNA showed an upward trend after 30 minutes of exercise, with a 40-fold increase after 90 minutes of exercise and a 100-fold increase to resting levels at the end of exercise (68). Plasma IL-6 levels have even been reported to increase 8,000-fold after a 264-kilometer marathon (69). Although short-term acute exercise increases circulating IL-6 levels, long-term exercise may decrease basal plasma IL-6 levels (70, 71).

It has been suggested that low-intensity exercise may lead to an increase in resting IL-6 levels, while high-intensity exercise may lead to a decrease in resting IL-6. Moderate-intensity (46-65%VO_2max_) causes IL-6 levels to begin rising after exercise or 3 hours after exercise, ranging from 1.33 to 4.20 times, while high-intensity (60%-89.12% VO2max) exercise increased even more, ranging from 1.59 to 26.79 times (72). It was shown by Mendham et al. that the highest IL-6 response occurred immediately after moderate-intensity exercise compared to low-intensity exercise and seemed to be independent of the type of exercise (73). Furthermore, it was shown by Cullen et al. that high-intensity interval exercise (HIIE, emphasizing an individual session) of the same duration induced greater plasma IL-6 levels than low-intensity exercise. However, the increase of IL-6 caused by short periods of high intensity exercise was small and did not seem to induce a response of anti-inflammatory factors (74). The opposite result was found in Leggate’s study, where HIIE induced higher IL-6 responses than continuous moderate-intensity exercise (75). In addition, higher-intensity exercise, such as High-Intensity Interval Training (HIIT), which emphasizes repeated structured training sessions, and Sprint Interval Training (SIT), a form of HIIT involving repeated all-out sprints with recovery periods, have been reported to elicit a greater metabolic response (76).

Studies on IL-6 concentration after exercise have been inconsistent. In different experimental studies, the difference in IL-6 concentration increase can be large, even at similar exercise intensity (74, 75, 77). This may be due to differences in the stage or severity of cancer cachexia and external environmental factors in different studies. In addition, exercise interventions used in different studies are diverse. These methodological differences hinder the definition of a uniform exercise standard. Fortunately, research suggests that a combination of exercise (aerobic and resistance) may improve inflammation and insulin resistance more than either type of exercise alone (76). Endurance exercise and resistance exercise improve muscle mass and function by regulating several key signaling pathways, such as Akt/mTOR, Akt/FOXO3a and adenosine monophosphate-activated protein kinase (AMPK), inhibiting E3 ubiquitin ligase, and mediating mitochondrial biosynthesis of AMPK/PGC-1α signaling pathway, but resistance exercise appears to have more obvious effects (78), which may prevent cancer-cachexia-induced muscle atrophy.

## 5 Exercise-induced IL-6 regulates cancer cachexia

**Figure 2:**
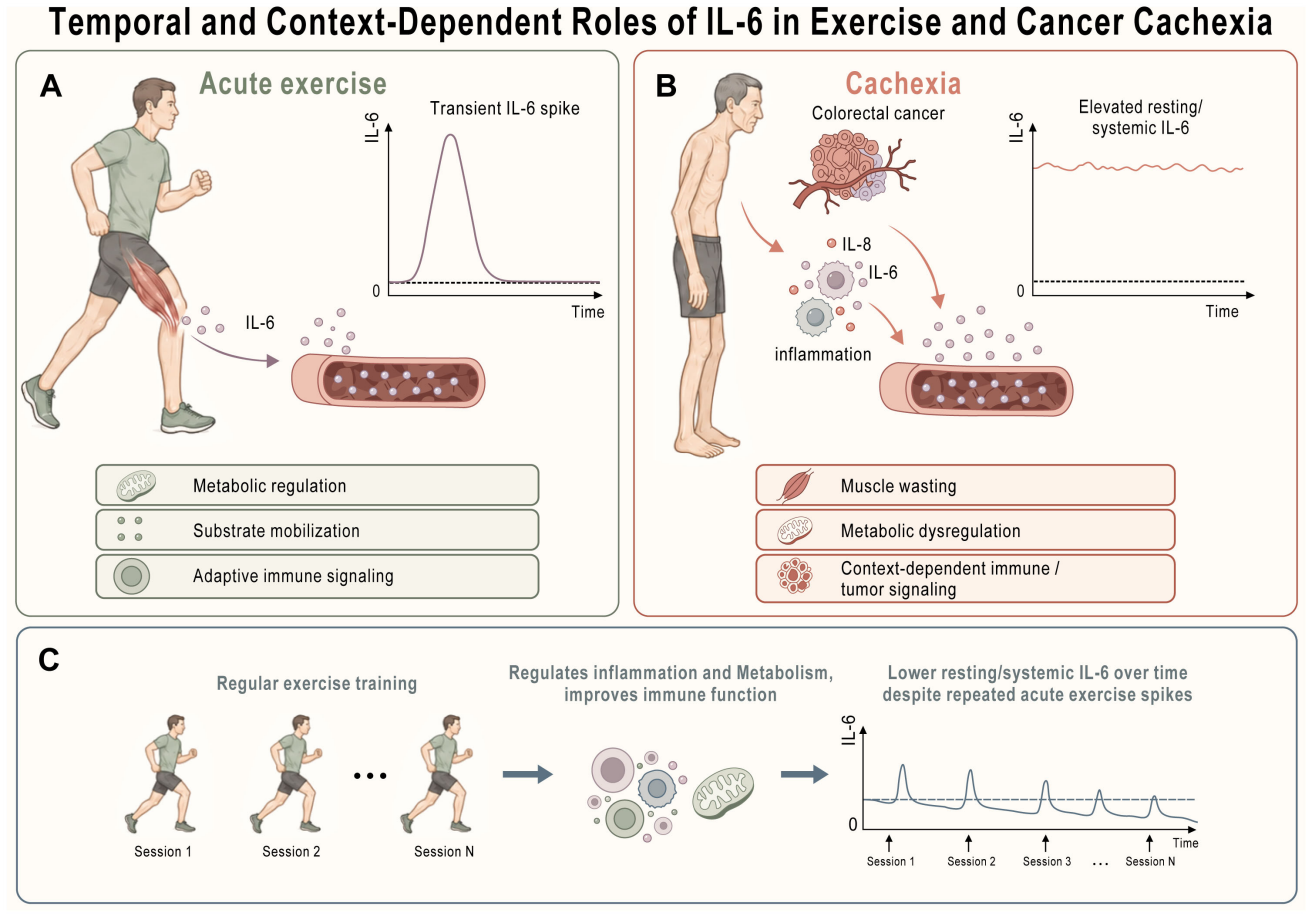
Temporal and context-dependent roles of IL-6 in exercise and cancer cachexia. **(A)** During acute exercise, contracting skeletal muscle releases IL-6 into the circulation, producing a transient IL-6 peak that contributes to metabolic regulation, substrate mobilization, and adaptive immune responses. **(B)** In cancer cachexia, persistently elevated resting/systemic IL-6 is associated with chronic inflammation, metabolic disturbance, muscle wasting, and context-dependent immune/tumor signaling. **(C)** Regular exercise training induces repeated short-lived IL-6 peaks after each exercise bout, progressively improving the inflammatory and metabolic milieu and lowering chronically elevated resting IL-6 over time. This temporal distinction helps reconcile the apparent paradox that exercise can acutely increase circulating IL-6 while reducing chronic systemic IL-6 with long-term training.

Exercise-induced IL-6 has been proposed to be associated with changes in inflammatory, metabolic, and immune pathways that may be relevant to the tumor immune environment and cancer cachexia risk (**Figure 2**).

### 5.1 Increased insulin sensitivity

Exercise can enhance skeletal muscle insulin sensitivity, making insulin more effective at promoting glucose uptake and utilization even at lower concentrations. During moderate-to-high intensity exercise, IL-6 has been implicated as one potential key mediator of this process, not only facilitating glucose uptake in muscle but also regulating endogenous glucose production (EGP) (79). Preclinical studies have shown that IL-6 knockout mice develop adult-onset obesity and insulin resistance, highlighting the critical role of IL-6 in metabolic homeostasis. In experimental models, during acute exercise, transient peaks of circulating IL-6 activate STAT3 signaling via muscle IL-6R. This activation subsequently upregulates GLUT4 expression during the recovery period, which correlates with enhanced skeletal muscle glucose uptake and improved insulin sensitivity. The effect is further enhanced through exercise-induced AMPK activation (80). In preclinical experiments, administration of IL-6 neutralizing antibodies prior to exercise abolishes this effect (80). Recombinant IL-6 at physiological post-exercise concentrations (50–100 µg/mL) can increase GLUT4 expression, whereas higher doses (100 µg/mL) do not further enhance GLUT4, indicating a clear dose- and time-dependent regulation.

IL-6–mediated ACC phosphorylation is AMPK-dependent and is associated with higher fatty acid oxidation (81, 82), lower intracellular lipid accumulation, and improved insulin sensitivity. This effect is independent of TNF-α, as IL-6 inhibits TNF-α–induced diacylglycerol (DAG) synthesis and attenuates its inhibitory effect on insulin action. Furthermore, IL-6 synergizes with insulin to reduce insulin-mediated suppression of fatty acid oxidation and decrease fatty acid esterification into triacylglycerol (TAG), thereby improving overall metabolic homeostasis (48). This effect is possible *in vitro*, *in vivo* and ex vivo settings. In ex vivo experiments, injection of 500 pg/mL IL-6 into isolated mouse gastrocnemius muscle elicited a significant effect, whereas 5000 pg/mL IL-6 did not produce further enhancement, indicating a dose-dependent window for IL-6 activity. In healthy men, intravenous infusion of recombinant IL-6 (Low-rhIL6: 30 μg/h, plasma IL-6 steady-state ≈140 pg/mL; High-rhIL6: 15 μg/h, plasma IL-6 ≈320 pg/mL) significantly increased plasma free fatty acid concentrations, and elevated glycerol release, while plasma triglycerides, insulin, epinephrine, and norepinephrine levels were largely unaffected (83). However, another study reported that during moderate-intensity exercise in healthy men (70% VO_2_peak, circulating IL-6 ≈9 pg/mL), fatty acid mobilization was markedly increased, with elevated glycerol release and concomitant rises in epinephrine, norepinephrine, and cortisol levels. In contrast, during low-intensity exercise (40% VO_2_peak, circulating IL-6 ≈3–4 pg/mL), infusion of recombinant IL-6 to achieve plasma IL-6 levels comparable to moderate-intensity exercise did not significantly enhance fatty acid mobilization or oxidation, nor did it alter insulin, epinephrine, or norepinephrine levels (84). These findings suggest that the metabolic actions of muscle-derived IL-6 cannot be fully recapitulated by recombinant IL-6 alone. Exercise-induced enhancement of fatty acid metabolism likely depends not only on circulating IL-6 but also on the associated hormonal milieu and potentially other yet-to-be-elucidated mechanisms, which warrant further investigation in future studies.

Ellingsgaard and colleagues reported that IL-6 regulates Glucagon-like peptide-1 (GLP-1) secretion in GLUTag L cells via two primary mechanisms. Muscle-derived IL-6 acutely enhanced GLP-1 secretion by directly increasing exocytosis, a process independent of Ca²^+^ currents and dependent on the JAK2-STAT3 signaling pathway. Chronic IL-6 exposure, on the other hand, increased GLP-1 biosynthesis and glucose uptake, rendering L cells more responsive to glucose and thereby augmenting glucose-stimulated GLP-1 secretion. In IL-6 knockout mice or mice administered IL-6 neutralizing antibodies prior to exercise, post-exercise increases in GLP-1 levels and insulin secretion were abolished, and inhibition of JAK2-STAT3 attenuated both glucose-induced GLP-1 secretion and its total content (85). A single injection of 400 ng IL-6 in mice (plasma IL-6 ≈ 552 ± 96 pg/mL, comparable to levels induced by exercise or high-fat diet) significantly enhanced GLP-1 regulation, whereas a low dose (4 ng) produced only modest effects. Subsequent randomized crossover studies in humans provided evidence consistent with IL-6 involvement in GLP-1 secretion via classical signaling. Additionally, IL-6R blockade with tocilizumab in humans inhibited exercise- or meal-induced increases in GLP-1 and insulin secretion. Similarly, in mice, IL-6 deficiency or neutralizing antibodies blocked exercise-induced GLP-1 elevation and improvements in insulin/glucose homeostasis (86), suggesting a potential role for IL-6 in exercise-associated GLP-1 changes.

Notably, circulating uncarboxylated and bioactive elevated osteocalcin may not only helps to enhance muscle glucose absorption during exercise, but also promotes IL-6 production and release (87). IL-6 is associated with higher levels of bioactive osteocalcin, which may correlate with adaptation to exercise (88).

### 5.2 Improvement of immune function

In the TME, some immune cells continuously produce IL-6, whereas circulating IL-6 increases only transiently after exercise (89) and participates in the regulation of immune responses (90).

Natural killer (NK) cells are a type of white blood cell in the immune system that is particularly sensitive to motor responses. During exercise, these cells can be rapidly recruited into tumors in preclinical models. This response may contribute to antitumor immune surveillance (91). Aerobic exercise lasting 20–60 minutes can activate the adrenergic pathway, whereby adrenaline mobilizes NK cells from the spleen and bone marrow into the circulation through β-adrenergic receptors. Chemokines such as CCL3, CXCL10, and CX3CL1 further facilitate NK-cell infiltration (92), thereby providing a prerequisite for the redistribution of muscle-derived IL-6 to tumors (93). Consistent with earlier studies (94), this suggests that adrenaline may act as an inducer of exercise-induced IL-6. Pedersen and colleagues observed in multiple murine tumor models that exercise mobilizes NK cells via both adrenergic- and IL-6–dependent mechanisms. Blockade of β-adrenergic receptors or IL-6 significantly reduced circulating NK cell numbers, whereas administration of recombinant IL-6 alone failed to recapitulate the effects of exercise.

Indeed, adrenaline plays a role in exercise-mediated immune cell responses (95–97). However, another study reported that during acute exercise in humans, IL-6R blockades reduced the recruitment of circulating NK cells but had no effect on T cells or neutrophils, and circulating levels of adrenaline and noradrenaline were largely unchanged (49). Mice treated with the combination of exercise training and doxorubicin showed increased levels of IL-6 and IL-5, accompanied by increased infiltration of NK cells and CD8 T cells (98). However, this effect was not observed in mice that did not use exercise training, which suggested that physical activity may contribute to immune modulation.

However, the immune effects of exercise-induced IL-6 are not uniformly beneficial but are closely dependent on exercise load. Acute high-intensity or prolonged endurance exercise can markedly increase circulating IL-6 levels, reaching 20–100 pg/mL, which is 10–100-fold higher than levels observed in healthy trained or untrained individuals, with accompanying increases in IL-10, IL-4, TNF-α, PGE_2_, and GA/CA (72). Such high-level cytokine responses, particularly when reaching 10–50-fold or more above baseline, may be associated with adverse effects such as tissue damage. Prolonged excessive exercise can induce tissue injury and promote the release of type 2 helper T cell (Th2)-associated cytokines, including IL-4 and IL-10, as well as other cytokines such as IL-6 and the pro-inflammatory cytokine TNF-α. IL-4 drives the differentiation of naïve CD4^+^ T cells toward the Th2 lineage and, together with IL-10, suppresses Th1 development and the secretion of key Th1 cytokines such as IFN-γ, thereby enhancing humoral immunity while inhibiting cell-mediated immunity (99). IL-6 may further amplify this process by stimulating CD4^+^ T cells to maintain IL-4 production and by activating stress hormone secretion, thereby reinforcing Th2 differentiation, suppressing Th1 responses, and aggravating immune imbalance. In addition, Th2-associated cytokines such as IL-5 and IL-13 can synergistically enhance Th2 responses, whereas the secretion of IL-12 and IFN-γ is suppressed, further exacerbating Th1/Th2 immune imbalance (99).

## 6 Exercise in colorectal cancer cachexia

Based on preclinical evidence from C26 murine models, various forms of exercise, including resistance training, endurance exercise and acute aerobic exercise—are associated with changes in markers related to CRC cachexia. Although a limited number of studies have reported positive effects of exercise in patients with CRC-associated cachexia (100–102), it should be noted that randomized controlled trials (RCTs) supporting this conclusion in humans remain scarce, and existing studies employ heterogeneous outcome measures for assessing CRC cachexia (**Table 1**).

**Table 1:** Effect of exercise on colorectal cancer and cachexia.

A. Preclinical animal models									
Model/population	Exercise modality	Protocol details			Primary cachexia/cancer outcomes	Inflammatory outcomes	Proposed mechanisms	Level of evidence	Ref.
		Frequency	Duration	Intensity					
Murine model: BALB/c mice	Combined resistance + endurance training	4 days/week	4 weeks pre-tumor implantation + 11 days during tumor growth	resistance 3×2 reps/load, endurance 25 min at 5–9 m·min^-^¹	TA mass partially preserved (~11–12%), grip strength largely preserved	Combined exercise reduced LC3B-II/I ratio; p62 remained high	Reduced excessive autophagy (LC3B-II/I), SDH activity preserved	Level 3	(103)
Murine model: CD2F1 mice with C26 colon carcinoma	Resistance training via sciatic nerve electrical stimulation	10 × 6 reps per session, 3 s contraction, 10 s rest	every other day, 8 sessions	maximal force; 100 Hz	attenuated EDL muscle wasting (+62%) and increased protein content (+25%);body weight decreased ~10%	pro-inflammatory cytokines and ubiquitin-proteasome activation	Increased protein synthesis;may act by inhibiting TNF-α signaling and/or reducing ubiquitin-proteasome activity.	Level 3	(104)
Combined preclinical model: BALB/c mice and C26 colon carcinoma cell culture	Endurance-like aerobic exercise	Daily	19 days	self-selected voluntary running	restored TA muscle mass and fiber CSA	Not directly measured	reduces p62 & LC3bII/LC3bI dysregulation, suppresses Atrogin-1 & MuRF1	Level 3	(105)
Combined preclinical model: CD2F1 mice and C26 colon carcinoma cell culture	High-intensity eccentric contractions via electrical stimulation (ECC-ES)	Every other day, 2 s contractions every 6 s, 4 sets of 5 reps	14 sessions total	100 Hz, 45 V	ECC-ES ameliorated gastrocnemius muscle atrophy and restored protein synthesis rate in C-26 mice	Not directly measured	Activation of mTORC1 signaling, increased phosphorylation of p70S6K/rpS6, inhibition of ubiquitin-proteasome pathway; autophagy (LC3B-II/I) increased but not inhibited	Level 3	(106)
Combined preclinical model: BALB/c mice and C26 colon carcinoma cell culture	Moderate-intensity aerobic (treadmill), resistance (ladder cage), or combined	TM: 5 days/week, RT: continuous, TM+RT: concurrent	8 weeks pre-implantation; TM 55 min/day, RT: progressive height sets	TM: 5% grade RT: progressive cage height increase forcing bipedal stance	TM: prevented gastrocnemius atrophy, preserved CSA of IIa/IIb fibers, attenuated grip loss; RT: preserved muscle mass and CSA, prevented grip strength decline; TM+RT: greatest preservation of muscle mass, CSA, and grip strength	All EX: normalized spleen mass, reduced plasma IL-6, indicating blunted systemic inflammation	Exercise-induced intermittent IL-6 release from muscle, anti-inflammatory effects, suppression of ubiquitin-proteasome pathway, uniform protection across MHC II fibers	Level 3	(107)
B. Human serum–based *in vitro*									
Ex vivo human study: serum from physically inactive adults (BMI ≥25) applied to LoVo colon cancer cell line	Acute moderate-intensity aerobic interval exercise on cycle ergometer	Acute, single session	6 × 5 min intervals + 2.5 min active recovery, total 30 min	60% heart rate reserve, RPE ≈13, power ≈94 W	Post-exercise serum reduced LoVo cell proliferation by 5.7% and γ-H2AX expression by 24.6%	Serum IL-6 increased 24.6% post-exercise; no changes in IL-8, TNF-α, IL-10, osteonectin, irisin, or oncostatin M	Exercise-induced IL-6 directly reduced LoVo proliferation and γ-H2AX in a dose-dependent manner, suggesting IL-6-mediated regulation of DNA damage and repair	Level 4	(108)
C. Clinical human studies									
Human clinical study: Stage I–III CRC survivors	Home-based moderate-intensity aerobic exercise	4.4 ± 0.3 sessions/week	12weeks, 30–60 min (5 min warm-up + 30–60 min aerobic + 5 min cool-down)	68.7% ± 1.1% of age-predicted max HR	No significant change in CTCs or tumor fraction from ctDNA over 12 weeks; higher exercise adherence correlated with reduced CTCs	No significant change in hs-CRP or IL-6 in overall cohort; subgroup with elevated baseline hs-CRP showed 35.5% reduction	potential anti-tumor effect may relate to exercise adherence and shear stress on CTCs rather than systemic inflammation	Level 2	(109)
Human clinical study: CRC survivors ≥1 month post-treatment	High-intensity interval exercise (HIIE) on cycle ergometer	acute single bout vs. 3×/week for 4 weeks	Acute session: 38 min total; 10 min warm-up (50–70% HRpeak) + 4 × 4 min at 85–95% HRpeak, 3 min active recovery between bouts; Short-term: same protocol 3×/week for 4 weeks (12 sessions)	85–95% HRpeak during intervals	Serum collected immediately post-acute HIIE reduced CaCo-2 and LoVo cell number at 24, 48, 72 h; 120 min post-exercise and short-term training serum had no effect	Acute HIIE: IL-6 (+44.8%), IL-8 (+24.7%), TNF-α (+15.2%) immediately post-exercise; levels returned to baseline by 120 min; short-term HIIE: no significant changes	Transient cytokine flux (IL-6, IL-8, TNF-α) following acute HIIE likely mediates reductions in colon cancer cell proliferation; repeated acute effects may contribute to long-term exercise benefits	Level 2	(110)

TA, Tibialis anterior; SDH, succinate dehydrogenase; EDL, extensor digitorum longus; CSA, cross-sectional area; ECC-ES, eccentric contractions via electrical stimulation; RPE, Rate of Perceived Exertion; TM, treadmill; RT, resistance training; EX, exercise; HR, Heart Rate; CTCs, circulating tumor cells; hs-CRP, high-sensitivity C-reactive protein; HIIE, High-intensity interval exercise.

Evidence suggests that resistance exercise is associated with larger increases in muscle mass and strength (111). Resistance exercise training has been reported to attenuate the rate of muscle loss and protein degradation in CRC mice (104). In a clinical trial, Tatebayashi and colleagues reported that the rate of protein synthesis in muscle from CRC patients was significantly reduced, but the expression of MuRF1 mRNA was enhanced instead. However, resistance exercise significantly activated the mTORC1 signaling molecule, which led to a certain increase in the rate of protein synthesis, while also limiting the expression of MuRF1 mRNA. These findings indicate that resistance training is associated with changes in pathways such as the ubiquitin-proteasome and mTORC1 signaling, which may relate to CRC-induced cachexia (106). Evidence suggests that endurance exercise may be more effective in maintaining and improving maximal aerobic capacity than resistance exercise (111). This effect may be closely related to mitochondrial content and function. CRC leads to reductions in both mitochondrial content and activity in mice, whereas endurance exercise training can partially preserve mitochondrial number (100) and function (103). This may promote mitochondrial biogenesis and respiration through enhanced PGC-1α expression (112). This process may contribute to the increase in muscle mass, decreasing the amount of muscle loss due to CRC and the severity of cachexia. Evidence suggests that muscle-derived IL-6 may reduce muscle protein synthesis by activating AMPK and inhibiting mTORC1 signaling, and chronic elevations of IL-6 are also known to stimulate AMPK (113). However, the increase in muscle-derived IL-6 during endurance exercise is transient and pulsatile, distinct from the sustained high levels observed in chronic inflammatory states. This transient and pulsatile pattern may support short-term AMPK activation and metabolic adaptation (114). AMPK is activated by reducing available energy and contraction in muscles, thereby inducing the body’s adaptation to endurance exercise (115). Notably, studies have found that AMPK signaling is markedly attenuated following short-term endurance training (116), and whether IL-6 serves as a necessary intermediate regulator in this context remains to be further investigated.

While both resistance and endurance exercise may ameliorate CRC-induced cachexia, their effects on different indicators appear to vary. Wood et al. reported that both forms of exercise were associated with reduced CRC-induced muscle weakness, with resistance exercise potentially having a greater effect (107). Plasma IL-6 concentration and splenomegaly were also measured in this experiment. Plasma IL-6 concentration and splenomegaly are important indicators of systemic inflammation (117). Both forms of exercise were observed to correlate with lower CRC-induced plasma IL-6 concentrations and splenomegaly in preclinical models, which may reflect a potential association with systemic inflammation (107). Although there are differences in the specific mechanisms of different exercise forms, the mechanism of action primarily relates to the ability to inhibit systemic inflammation, which is manifested by effectively reducing IL-6 levels.

Furthermore, muscle-derived IL-6 has been linked to anti-inflammatory and metabolic protective effects by upregulating IL-1RA and IL-10, while suppressing TNF-α through a STAT3-dependent mechanism (118). *In vitro* studies using U937 cells treated with recombinant IL-6 showed inhibitory effects beginning at 3 ng/mL, with maximal suppression (approximately 60–95% reduction in TNF-α) observed at 20–40 ng/mL. Similarly, recombinant IL-6 treatment of human peripheral mononuclear cells inhibited lipopolysaccharide (LPS)-induced TNF-α production, with low doses (0.3 ng/mL) initiating effects and moderate doses (20–40 ng/mL) producing the strongest inhibition. *In vivo*, intraperitoneal injection of 10 pg recombinant IL-6 in healthy mice significantly suppressed plasma TNF-α levels following Bacille Calmette-Guérin activation and LPS stimulation, and this inhibition of TNF-α production was independent of adrenaline (119).

However, clinical studies have shown that exercise can transiently increase IL-6 levels. Starkie et al. found that cycling was accompanied by increased circulating IL-6 and reduced endotoxin-induced TNF-α secretion (120). As the duration of exercise becomes longer, the IL-6 concentration appears to increase correspondingly (121). In addition, the effects of exercise on IL-6 and TNF-α levels also depend on the frequency and intensity of exercise (122). Orange and colleagues found that serum collected after acute aerobic exercise suppressed the proliferation of LoVo CRC cells, with proliferation decreasing as IL-6 concentrations increased. *In vitro*, recombinant IL-6 (0.1–100 pg/mL) dose-dependently recapitulated the anti-proliferative effects of exercise serum, suggesting that IL-6 may serve as a key contributor. However, IL-6 neutralizing antibodies were not applied to the exercise serum in this study, so the potential contribution of other serum factors to proliferation inhibition cannot be entirely excluded. Measured serum factors, including IL-8, TNF-α, osteonectin, and OSM—did not significantly change following exercise. Nevertheless, exercise serum may contain thousands of metabolites, peptides, and RNA molecules that could also participate in CRC inhibition, although current evidence is consistent with a possible contribution of IL-6. IL-6 may contribute to exercise serum-mediated suppression of CRC cell proliferation (108). However, current evidence does not establish IL-6 as the sole mediator or define a clinically applicable anti-tumor concentration cutoff.

## 7 Discussion and conclusion

The role of IL-6 in CRC and colorectal cancer–related cachexia is context-dependent, with its effects determined by signaling mode, cellular source, temporal dynamics, tumor context, and other interacting factors. In cancer cachexia, persistently elevated resting IL-6 is generally mediated via trans-signaling and is commonly associated with muscle wasting, metabolic dysregulation, and pro-tumorigenic signaling. By contrast, acute exercise induces transient increases in IL-6 primarily through classical signaling pathways, potentially supporting metabolic regulation, substrate mobilization, and immune adaptation (123). Regular exercise training, through improvements in inflammatory and metabolic milieu and engagement of immune modulation, gradually lowers chronically elevated resting IL-6 over time. Compared with other pro-inflammatory cytokines, such as TNF-α and IL-8, muscle-derived IL-6 appears to be closely linked to CRC interventions and exercise-related outcomes, functioning as an important context-dependent regulatory mediator.

Current evidence indicates that in healthy individuals, plasma IL−6 concentrations are typically very low (approximately 0–7 pg/mL) (124). However, IL−6 levels can increase markedly in acute inflammatory or infectious states (125). In conditions of severe infection such as sepsis, plasma IL−6 levels exceeding approximately 1000 pg/mL are often indicative of uncontrolled inflammation and are associated with poor prognosis, warranting timely clinical intervention (126). Nevertheless, this threshold is primarily used for clinical assessment of inflammatory severity rather than defining an inherent harmful concentration of IL−6 itself. Based on the evidence reviewed above, exercise-induced IL-6 effects may vary with concentration, with lower basal levels potentially contributing to homeostasis, moderate acute elevations possibly supporting metabolic and immune regulation, and chronically high or sustained elevations being associated with inflammation and muscle wasting. IL-6 may also exhibit a plateau-like effect, where moderate increases could confer benefits, whereas excessive or prolonged elevations might lead to adverse outcomes. However, precise quantitative thresholds for IL-6 dose–response relationships have not been established (48, 80, 99), as the physiological and pathological effects of IL-6 concentrations differ substantially across animal models, *in vitro* experiments, and human studies. Mechanistic investigations into the potential plateau effect of IL-6 remain limited, and any classification of IL-6 effects should be considered speculative and hypothesis-generating rather than definitive.

Exercise interventions for patients with CRC and CRC-related cachexia should be carefully designed to minimize inflammatory and metabolic risks. Mechanistic insights into exercise-induced IL-6 signaling primarily come from physiological and preclinical studies (20, 90). In cachexia animal models, moderate exercise has been reported to attenuate muscle wasting and modulate IL-6–related pathways (107), while clinical evidence in CRC populations remains limited. Nevertheless, studies in CRC survivors suggest that moderate-intensity aerobic exercise may influence inflammatory biomarkers, including IL-6 (127). Therefore, exercise interventions should be regarded as multifactorial and individualized supportive strategies that could be explored in future research, rather than as clinically validated prescriptions.

Clinical monitoring remains essential and may include body weight, muscle mass, physical performance, fatigue, treatment-related toxicities, and biochemical markers of inflammation and muscle damage. Circulating IL-6 may serve as a potential biomarker, but its interpretation should be cautious and integrated with multidimensional indicators such as CRP, creatine kinase, lactate dehydrogenase, leukocyte profiles, nutritional status, and clinical symptoms. For patients with advanced CRC or severe cachexia, low-intensity, supervised exercise approaches could be considered to preserve muscle mass, functional capacity, and daily activity while avoiding excessive physiological stress.

It should be emphasized that the benefits of exercise are unlikely to be mediated predominantly by IL-6 alone. Instead, they involve coordinated changes in skeletal muscle metabolism, AMPK signaling, catecholamine-mediated immune cell mobilization, insulin sensitivity, mitochondrial function, systemic inflammatory remodeling, and multiple myokines and metabolites. It is important to highlight that exercise-induced IL-6 levels operate within the complex physiological and pathophysiological interplay of cancer cachexia, where they can only effectively slow disease progression and reduce muscle wasting, but not reverse established depletion. Thus, serum IL-6 dynamics should currently be considered a candidate biomarker or mechanistic readout, rather than a validated basis for exercise prescription. Future studies are needed to determine whether IL-6 dynamics can reliably predict exercise responses, stratify risk, or guide individualized exercise interventions.

It should be noted that this article is a narrative review. The included studies comprised human intervention trials, observational studies, animal models, and *in vitro* experiments. Articles that were irrelevant or lacked sufficient methodological detail were excluded. Mechanistic insights derived from preclinical studies were primarily used to support hypotheses rather than to establish causal relationships. The conclusions of this review should be regarded as exploratory and hypothesis-generating. Studies were selected based on their relevance to IL-6 signaling, exercise, and colorectal cancer–related cachexia. For clinical interpretation, human interventional studies were prioritized, followed by observational studies, animal models, and *in vitro* mechanistic studies.

## References

[1] Sung H Ferlay J Siegel RL Laversanne M Soerjomataram I Jemal A . Global cancer statistics 2020: GLOBOCAN estimates of incidence and mortality worldwide for 36 cancers in 185 countries. CA Cancer J Clin. (2021) 71:209–49. doi: 10.3322/caac.21660 33538338

[2] Bray F Jemal A Grey N Ferlay J Forman D . Global cancer transitions according to the Human Development Index (2008-2030): a population-based study. Lancet Oncol. (2012) 13:790–801. doi: 10.1016/s1470-2045(12)70211-5 22658655

[3] Qiu C Shi W Wu H Zou S Li J Wang D . Identification of molecular subtypes and a prognostic signature based on inflammation-related genes in colon adenocarcinoma. Front Immunol. (2021) 12:769685. doi: 10.3389/fimmu.2021.769685 35003085 PMC8733947

[4] Schäfer M Werner S . Cancer as an overhealing wound: an old hypothesis revisited. Nat Rev Mol Cell Biol. (2008) 9:628–38. doi: 10.1038/nrm2455 18628784

[5] Berster JM Göke B . Type 2 diabetes mellitus as risk factor for colorectal cancer. Arch Physiol Biochem. (2008) 114:84–98. doi: 10.1080/13813450802008455 18465362

[6] Pongratz G Straub RH . The sympathetic nervous response in inflammation. Arthritis Res Ther. (2014) 16:504. doi: 10.1186/s13075-014-0504-2 25789375 PMC4396833

[7] Fearon KC Voss AC Hustead DS . Definition of cancer cachexia: effect of weight loss, reduced food intake, and systemic inflammation on functional status and prognosis. Am J Clin Nutr. (2006) 83:1345–50. doi: 10.1093/ajcn/83.6.1345 16762946

[8] Vagnildhaug OM Balstad TR Almberg SS Brunelli C Knudsen AK Kaasa S . A cross-sectional study examining the prevalence of cachexia and areas of unmet need in patients with cancer. Support Care Cancer. (2018) 26:1871–80. doi: 10.1007/s00520-017-4022-z 29274028

[9] Kasprzak A . The role of tumor microenvironment cells in colorectal cancer (CRC) cachexia. Int J Mol Sci. (2021) 22(4):1565. doi: 10.3390/ijms22041565 33557173 PMC7913937

[10] Murakami M Kamimura D Hirano T . Pleiotropy and specificity: insights from the interleukin 6 family of cytokines. Immunity. (2019) 50:812–31. doi: 10.1016/j.immuni.2019.03.027 30995501

[11] Hojman P Brolin C Nørgaard-Christensen N Dethlefsen C Lauenborg B Olsen CK . IL-6 release from muscles during exercise is stimulated by lactate-dependent protease activity. Am J Physiol Endocrinol Metab. (2019) 316:E940–7. doi: 10.1152/ajpendo.00414.2018 30779630

[12] Iwase S Murakami T Saito Y Nakagawa K . Steep elevation of blood interleukin-6 (IL-6) associated only with late stages of cachexia in cancer patients. Eur Cytokine Netw. (2004) 15:312–6. 15627639

[13] Feliciano EMC Kroenke CH Meyerhardt JA Prado CM Bradshaw PT Kwan ML . Association of systemic inflammation and sarcopenia with survival in nonmetastatic colorectal cancer: results from the C SCANS study. JAMA Oncol. (2017) 3:e172319. doi: 10.1001/jamaoncol.2017.2319 28796857 PMC5824285

[14] Steensberg A van Hall G Osada T Sacchetti M Saltin B Klarlund Pedersen B . Production of interleukin-6 in contracting human skeletal muscles can account for the exercise-induced increase in plasma interleukin-6. J Physiol. (2000) 529 Pt 1:237–42. doi: 10.1111/j.1469-7793.2000.00237.x 11080265 PMC2270169

[15] Pedersen BK . Exercise-induced myokines and their role in chronic diseases. Brain Behav Immun. (2011) 25:811–6. doi: 10.1016/j.bbi.2011.02.010 21354469

[16] Whitham M Parker BL Friedrichsen M Hingst JR Hjorth M Hughes WE . Extracellular vesicles provide a means for tissue crosstalk during exercise. Cell Metab. (2018) 27:237–251.e4. doi: 10.1016/j.cmet.2017.12.001 29320704

[17] Moore SC Lee IM Weiderpass E Campbell PT Sampson JN Kitahara CM . Association of leisure-time physical activity with risk of 26 types of cancer in 1.44 million adults. JAMA Intern Med. (2016) 176:816–25. doi: 10.1001/jamainternmed.2016.1548 27183032 PMC5812009

[18] Daou HN . Exercise as an anti-inflammatory therapy for cancer cachexia: a focus on interleukin-6 regulation. Am J Physiol Regul Integr Comp Physiol. (2020) 318:R296–310. doi: 10.1152/ajpregu.00147.2019 31823669

[19] Brown JC Rickels MR Troxel AB Zemel BS Damjanov N Ky B . Dose-response effects of exercise on insulin among colon cancer survivors. Endocr Relat Cancer. (2018) 25:11–9. doi: 10.1530/erc-17-0377 29018055 PMC5736434

[20] Pedersen BK Febbraio MA . Muscle as an endocrine organ: focus on muscle-derived interleukin-6. Physiol Rev. (2008) 88:1379–406. doi: 10.1152/physrev.90100.2007 18923185

[21] Tanji Y Furukawa K Haruki K Taniai T Onda S Tsunematsu M . Significant impact of cachexia index on the outcomes after hepatic resection for colorectal liver metastases. Ann Gastroenterol Surg. (2022) 6:804–12. doi: 10.1002/ags3.12578 36338593 PMC9628226

[22] Lopez-Pedrosa JM Camprubi-Robles M Guzman-Rolo G Lopez-Gonzalez A Garcia-Almeida JM Sanz-Paris A . The vicious cycle of type 2 diabetes mellitus and skeletal muscle atrophy: clinical, biochemical, and nutritional bases. Nutrients. (2024) 16(2). doi: 10.3390/nu16010172 38202001 PMC10780454

[23] Senn JJ Klover PJ Nowak IA Zimmers TA Koniaris LG Furlanetto RW . Suppressor of cytokine signaling-3 (SOCS-3), a potential mediator of interleukin-6-dependent insulin resistance in hepatocytes. J Biol Chem. (2003) 278:13740–6. doi: 10.1074/jbc.m210689200 12560330

[24] Xie H Gong Y Kuang J Yan L Ruan G Tang S . Computed tomography-determined sarcopenia is a useful imaging biomarker for predicting postoperative outcomes in elderly colorectal cancer patients. Cancer Res Treat. (2020) 52:957–72. doi: 10.4143/crt.2019.695 32311863 PMC7373859

[25] Aimo A Saccaro LF Borrelli C Fabiani I Gentile F Passino C . The ergoreflex: how the skeletal muscle modulates ventilation and cardiovascular function in health and disease. Eur J Heart Fail. (2021) 23:1458–67. doi: 10.1002/ejhf.2298 34268843 PMC9292527

[26] Perazza LR Brown-Borg HM Thompson LV . Physiological systems in promoting frailty. Compr Physiol. (2022) 12:3575–620. doi: 10.1002/j.2040-4603.2022.tb00224.x 35578945 PMC9531553

[27] Li CW Yu K Shyh-Chang N Jiang Z Liu T Ma S . Pathogenesis of sarcopenia and the relationship with fat mass: descriptive review. J Cachexia Sarcopenia Muscle. (2022) 13:781–94. doi: 10.1002/jcsm.12901 35106971 PMC8977978

[28] Guzik TJ Korbut R Adamek-Guzik T . Nitric oxide and superoxide in inflammation and immune regulation. J Physiol Pharmacol. (2003) 54:469–87. 14726604

[29] Puppa MJ White JP Velázquez KT Baltgalvis KA Sato S Baynes JW . The effect of exercise on IL-6-induced cachexia in the Apc (Min/+) mouse. J Cachexia Sarcopenia Muscle. (2012) 3:117–37. doi: 10.1007/s13539-011-0047-1 PMC3374019 22476915

[30] Taga T Hibi M Hirata Y Yamasaki K Yasukawa K Matsuda T . Interleukin-6 triggers the association of its receptor with a possible signal transducer, gp130. Cell. (1989) 58:573–81. doi: 10.1016/0092-8674(89)90438-8 2788034

[31] Zegeye MM Lindkvist M Fälker K Kumawat AK Paramel G Grenegård M . Activation of the JAK/STAT3 and PI3K/AKT pathways are crucial for IL-6 trans-signaling-mediated pro-inflammatory response in human vascular endothelial cells. Cell Commun Signal. (2018) 16:55. doi: 10.1186/s12964-018-0268-4 30185178 PMC6125866

[32] Jones GW McLoughlin RM Hammond VJ Parker CR Williams JD Malhotra R . Loss of CD4+ T cell IL-6R expression during inflammation underlines a role for IL-6 trans signaling in the local maintenance of Th17 cells. J Immunol. (2010) 184:2130–9. doi: 10.4049/jimmunol.0901528 20083667

[33] Schumacher N Meyer D Mauermann A von der Heyde J Wolf J Schwarz J . Shedding of endogenous interleukin-6 receptor (IL-6R) is governed by A disintegrin and metalloproteinase (ADAM) proteases while a full-length IL-6R isoform localizes to circulating microvesicles. J Biol Chem. (2015) 290:26059–71. doi: 10.1074/jbc.m115.649509 26359498 PMC4646259

[34] Gnosa SP Puig Blasco L Piotrowski KB Freiberg ML Savickas S Madsen DH . ADAM17-mediated EGFR ligand shedding directs macrophage-promoted cancer cell invasion. JCI Insight. (2022) 7(18). doi: 10.1172/jci.insight.155296 35998057 PMC9675555

[35] Lamertz L Rummel F Polz R Baran P Hansen S Waetzig GH . Soluble gp130 prevents interleukin-6 and interleukin-11 cluster signaling but not intracellular autocrine responses. Sci Signal. (2018) 11(550). doi: 10.1126/scisignal.aar7388 30279168

[36] Bihl MP Heinimann K Rüdiger JJ Eickelberg O Perruchoud AP Tamm M . Identification of a novel IL-6 isoform binding to the endogenous IL-6 receptor. Am J Respir Cell Mol Biol. (2002) 27:48–56. doi: 10.1165/ajrcmb.27.1.4637 12091245

[37] Alberti L Bachelot T Duc A Biota C Blay JY . A spliced isoform of interleukin 6 mRNA produced by renal cell carcinoma encodes for an interleukin 6 inhibitor. Cancer Res. (2005) 65:2–5. doi: 10.1158/0008-5472.2.65.1 15665272

[38] Annibalini G Guescini M Agostini D Matteis RD Sestili P Tibollo P . The expression analysis of mouse interleukin-6 splice variants argued against their biological relevance. BMB Rep. (2012) 45:32–7. doi: 10.5483/bmbrep.2012.45.1.32 22281010

[39] Aliyu M Zohora FT Anka AU Ali K Maleknia S Saffarioun M . Interleukin-6 cytokine: an overview of the immune regulation, immune dysregulation, and therapeutic approach. Int Immunopharmacol. (2022) 111:109130. doi: 10.1016/j.intimp.2022.109130 35969896

[40] Taher MY Davies DM Maher J . The role of the interleukin (IL)-6/IL-6 receptor axis in cancer. Biochem Soc Trans. (2018) 46:1449–62. doi: 10.1042/bst20180136 30467123

[41] Gabay C . Interleukin-6 and chronic inflammation. Arthritis Res Ther. (2006) 8 Suppl 2:S3. doi: 10.1186/ar1917 PMC3226076 16899107

[42] Watson C Whittaker S Smith N Vora AJ Dumonde DC Brown KA . IL-6 acts on endothelial cells to preferentially increase their adherence for lymphocytes. Clin Exp Immunol. (1996) 105:112–9. doi: 10.1046/j.1365-2249.1996.d01-717.x 8697617 PMC2200481

[43] Hurst SM Wilkinson TS McLoughlin RM Jones S Horiuchi S Yamamoto N . Il-6 and its soluble receptor orchestrate a temporal switch in the pattern of leukocyte recruitment seen during acute inflammation. Immunity. (2001) 14:705–13. doi: 10.1016/s1074-7613(01)00151-0 11420041

[44] Kaplanski G Marin V Montero-Julian F Mantovani A Farnarier C . IL-6: a regulator of the transition from neutrophil to monocyte recruitment during inflammation. Trends Immunol. (2003) 24:25–9. doi: 10.1016/s1471-4906(02)00013-3 12495721

[45] Fadok VA Bratton DL Konowal A Freed PW Westcott JY Henson PM . Macrophages that have ingested apoptotic cells *in vitro* inhibit proinflammatory cytokine production through autocrine/paracrine mechanisms involving TGF-beta, PGE2, and PAF. J Clin Invest. (1998) 101:890–8. doi: 10.1172/jci1112 9466984 PMC508637

[46] Gleeson M Bishop NC Stensel DJ Lindley MR Mastana SS Nimmo MA . The anti-inflammatory effects of exercise: mechanisms and implications for the prevention and treatment of disease. Nat Rev Immunol. (2011) 11:607–15. doi: 10.1038/nri3041 21818123

[47] Plomgaard P Penkowa M Pedersen BK . Fiber type specific expression of TNF-alpha, IL-6 and IL-18 in human skeletal muscles. Exerc Immunol Rev. (2005) 11:53–63. 16385844

[48] Bruce CR Dyck DJ . Cytokine regulation of skeletal muscle fatty acid metabolism: effect of interleukin-6 and tumor necrosis factor-alpha. Am J Physiol Endocrinol Metab. (2004) 287:E616–21. doi: 10.1152/ajpendo.00150.2004 15149950

[49] Bay ML Heywood S Wedell-Neergaard AS Schauer T Lehrskov LL Christensen RH . Human immune cell mobilization during exercise: effect of IL-6 receptor blockade. Exp Physiol. (2020) 105:2086–98. doi: 10.1113/ep088864 33006190

[50] Taniguchi K Karin M . IL-6 and related cytokines as the critical lynchpins between inflammation and cancer. Semin Immunol. (2014) 26:54–74. doi: 10.1016/j.smim.2014.01.001 24552665

[51] Ning Y Lenz HJ . Targeting IL-8 in colorectal cancer. Expert Opin Ther Targets. (2012) 16:491–7. doi: 10.1517/14728222.2012.677440 22494524

[52] Biasi F Guina T Maina M Nano M Falcone A Aroasio E . Progressive increase of matrix metalloprotease-9 and interleukin-8 serum levels during carcinogenic process in human colorectal tract. PloS One. (2012) 7:e41839. doi: 10.1371/journal.pone.0041839 22848630 PMC3405044

[53] Nagasaki T Hara M Nakanishi H Takahashi H Sato M Takeyama H . Interleukin-6 released by colon cancer-associated fibroblasts is critical for tumour angiogenesis: anti-interleukin-6 receptor antibody suppressed angiogenesis and inhibited tumour-stroma interaction. Br J Cancer. (2014) 110:469–78. doi: 10.1038/bjc.2013.748 24346288 PMC3899773

[54] Rogler G Gelbmann CM Vogl D Brunner M Schölmerich J Falk W . Differential activation of cytokine secretion in primary human colonic fibroblast/myofibroblast cultures. Scand J Gastroenterol. (2001) 36:389–98. doi: 10.1080/00365520119240 11336164

[55] Matsumoto S Hara T Mitsuyama K Yamamoto M Tsuruta O Sata M . Essential roles of IL-6 trans-signaling in colonic epithelial cells, induced by the IL-6/soluble-IL-6 receptor derived from lamina propria macrophages, on the development of colitis-associated premalignant cancer in a murine model. J Immunol. (2010) 184:1543–51. doi: 10.4049/jimmunol.0801217 20042582

[56] Huynh PT Beswick EJ Coronado YA Johnson P O'Connell MR Watts T . CD90(+) stromal cells are the major source of IL-6, which supports cancer stem-like cells and inflammation in colorectal cancer. Int J Cancer. (2016) 138:1971–81. doi: 10.1002/ijc.29939 PMC4865268 26595254

[57] Tseng-Rogenski SS Hamaya Y Choi DY Carethers JM . Interleukin 6 alters localization of hMSH3, leading to DNA mismatch repair defects in colorectal cancer cells. Gastroenterology. (2015) 148:579–89. doi: 10.1053/j.gastro.2014.11.027 25461668 PMC4339542

[58] Huang J Xiao R Shi S Li Q Li M Xiao M . Circulating IL6 is involved in the infiltration of M2 macrophages and CD8+ T cells. Sci Rep. (2025) 15:8681. doi: 10.1038/s41598-025-92817-9 40082587 PMC11906812

[59] Eisenthal A Kashtan H Rabau M Ramakrishna V Chaitchik S Skornick Y . Antitumor effects of recombinant interleukin-6 expressed in eukaryotic cells. Cancer Immunol Immunother. (1993) 36:101–7. doi: 10.1007/bf01754409 8425207 PMC11038442

[60] He T Hu C Li S Fan Y Xie F Sun X . The role of CD8(+) T-cells in colorectal cancer immunotherapy. Heliyon. (2024) 10:e33144. doi: 10.1016/j.heliyon.2024.e33144 39005910 PMC11239598

[61] Hailemichael Y Johnson DH Abdel-Wahab N Foo WC Bentebibel SE Daher M . Interleukin-6 blockade abrogates immunotherapy toxicity and promotes tumor immunity. Cancer Cell. (2022) 40:509–523.e6. doi: 10.1016/j.annonc.2021.10.090 35537412 PMC9221568

[62] Fischer CP . Interleukin-6 in acute exercise and training: what is the biological relevance? Exerc Immunol Rev. (2006) 12:6–33. 17201070

[63] Finsterer J . Biomarkers of peripheral muscle fatigue during exercise. BMC Musculoskelet Disord. (2012) 13:218. doi: 10.1186/1471-2474-13-218 23136874 PMC3534479

[64] Ostrowski K Hermann C Bangash A Schjerling P Nielsen JN Pedersen BK . A trauma-like elevation of plasma cytokines in humans in response to treadmill running. J Physiol. (1998) 513:889–94. doi: 10.1163/18763316-03903005 PMC2231318 9824725

[65] Toft AD Falahati A Steensberg A . Source and kinetics of interleukin-6 in humans during exercise demonstrated by a minimally invasive model. Eur J Appl Physiol. (2011) 111:1351–9. doi: 10.1007/s00421-010-1755-5 21153418

[66] Baran P Hansen S Waetzig GH Akbarzadeh M Lamertz L Huber HJ . The balance of interleukin (IL)-6, IL-6·soluble IL-6 receptor (sIL-6R), and IL-6·sIL-6R·sgp130 complexes allows simultaneous classic and trans-signaling. J Biol Chem. (2018) 293:6762–75. doi: 10.1074/jbc.ra117.001163 29559558 PMC5936821

[67] Lippitz BE . Cytokine patterns in patients with cancer: a systematic review. Lancet Oncol. (2013) 14:e218–28. doi: 10.1016/s1470-2045(12)70582-x 23639322

[68] Keller C Steensberg A Pilegaard H Osada T Saltin B Pedersen BK . Transcriptional activation of the IL-6 gene in human contracting skeletal muscle: influence of muscle glycogen content. FASEB J. (2001) 15:2748–50. doi: 10.1096/fj.01-0507fje 11687509

[69] Margeli A Skenderi K Tsironi M Hantzi E Matalas AL Vrettou C . Dramatic elevations of interleukin-6 and acute-phase reactants in athletes participating in the ultradistance foot race spartathlon: severe systemic inflammation and lipid and lipoprotein changes in protracted exercise. J Clin Endocrinol Metab. (2005) 90:3914–8. doi: 10.1210/jc.2004-2346 15855262

[70] Shalamzari SA Agha-Alinejad H Alizadeh S Shahbazi S Khatib ZK Kazemi A . The effect of exercise training on the level of tissue IL-6 and vascular endothelial growth factor in breast cancer bearing mice. Iran J Basic Med Sci. (2014) 17:231–58. doi: 10.22038/ijbms.2014.2579 PMC4046231 24904714

[71] Zylstra J Whyte GP Beckmann K Pate J Santaolalla A Gervais-Andre L . Exercise prehabilitation during neoadjuvant chemotherapy may enhance tumour regression in oesophageal cancer: results from a prospective non-randomised trial. Br J Sports Med. (2022) 56:402–9. doi: 10.1136/bjsports-2021-104243 35105604

[72] Cerqueira É Marinho DA Neiva HP Lourenço O . Inflammatory effects of high and moderate intensity exercise-A systematic review. Front Physiol. (2019) 10:1550. doi: 10.3389/fphys.2019.01550 31992987 PMC6962351

[73] Mendham AE Donges CE Liberts EA Duffield R . Effects of mode and intensity on the acute exercise-induced IL-6 and CRP responses in a sedentary, overweight population. Eur J Appl Physiol. (2011) 111:1035–45. doi: 10.1007/s00421-010-1724-z 21088973

[74] Cullen T Thomas AW Webb R Hughes MG . Interleukin-6 and associated cytokine responses to an acute bout of high-intensity interval exercise: the effect of exercise intensity and volume. Appl Physiol Nutr Metab. (2016) 41:803–8. doi: 10.1139/apnm-2015-0640 27377137

[75] Leggate M Nowell MA Jones SA Nimmo MA . The response of interleukin-6 and soluble interleukin-6 receptor isoforms following intermittent high intensity and continuous moderate intensity cycling. Cell Stress Chaperones. (2010) 15:827–33. doi: 10.1007/s12192-010-0192-z 20396982 PMC3024071

[76] Lee DH de Rezende LFM Eluf-Neto J Wu K Tabung FK Giovannucci EL . Association of type and intensity of physical activity with plasma biomarkers of inflammation and insulin response. Int J Cancer. (2019) 145:360–9. doi: 10.1002/ijc.32111 30614528 PMC6525061

[77] Leggate M Carter WG Evans MJ Vennard RA Sribala-Sundaram S Nimmo MA . Determination of inflammatory and prominent proteomic changes in plasma and adipose tissue after high-intensity intermittent training in overweight and obese males. J Appl Physiol (1985). (2012) 112:1353–60. doi: 10.1152/japplphysiol.01080.2011 22267387 PMC3331586

[78] Zeng Z Liang J Wu L Zhang H Lv J Chen N . Exercise-induced autophagy suppresses sarcopenia through akt/mTOR and akt/foxO3a signal pathways and AMPK-mediated mitochondrial quality control. Front Physiol. (2020) 11:583478. doi: 10.3389/fphys.2020.583478 33224037 PMC7667253

[79] Kreisman SH Halter JB Vranic M Marliss EB . Combined infusion of epinephrine and norepinephrine during moderate exercise reproduces the glucoregulatory response of intense exercise. Diabetes. (2003) 52:1347–54. doi: 10.2337/diabetes.52.6.1347 12765943

[80] Ikeda SI Tamura Y Kakehi S Sanada H Kawamori R Watada H . Exercise-induced increase in IL-6 level enhances GLUT4 expression and insulin sensitivity in mouse skeletal muscle. Biochem Biophys Res Commun. (2016) 473:947–52. doi: 10.1016/j.bbrc.2016.03.159 27040770

[81] Carey AL Steinberg GR Macaulay SL Thomas WG Holmes AG Ramm G . Interleukin-6 increases insulin-stimulated glucose disposal in humans and glucose uptake and fatty acid oxidation *in vitro* via AMP-activated protein kinase. Diabetes. (2006) 55:2688–97. doi: 10.2337/db05-1404 17003332

[82] Kahn BB Alquier T Carling D Hardie DG . AMP-activated protein kinase: ancient energy gauge provides clues to modern understanding of metabolism. Cell Metab. (2005) 1:15–25. doi: 10.1016/j.cmet.2004.12.003 16054041

[83] van Hall G Steensberg A Sacchetti M Fischer C Keller C Schjerling P . Interleukin-6 stimulates lipolysis and fat oxidation in humans. J Clin Endocrinol Metab. (2003) 88:3005–10. doi: 10.1210/jc.2002-021687 12843134

[84] Hiscock N Fischer CP Sacchetti M van Hall G Febbraio MA Pedersen BK . Recombinant human interleukin-6 infusion during low-intensity exercise does not enhance whole body lipolysis or fat oxidation in humans. Am J Physiol Endocrinol Metab. (2005) 289:E2–7. doi: 10.1152/ajpendo.00274.2004 15741245

[85] Ellingsgaard H Hauselmann I Schuler B Habib AM Baggio LL Meier DT . Interleukin-6 enhances insulin secretion by increasing glucagon-like peptide-1 secretion from L cells and alpha cells. Nat Med. (2011) 17:1481–9. doi: 10.1038/nm.2513 22037645 PMC4286294

[86] Ellingsgaard H Seelig E Timper K Coslovsky M Soederlund L Lyngbaek MP . GLP-1 secretion is regulated by IL-6 signalling: a randomised, placebo-controlled study. Diabetologia. (2020) 63:362–73. doi: 10.1007/s00125-019-05045-y 31796986

[87] Mera P Laue K Ferron M Confavreux C Wei J Galán-Díez M . Osteocalcin signaling in myofibers is necessary and sufficient for optimum adaptation to exercise. Cell Metab. (2016) 23:1078–92. doi: 10.1016/j.cmet.2016.12.003 27304508 PMC4910629

[88] Battafarano G Rossi M Marampon F Minisola S Del Fattore A . Bone control of muscle function. Int J Mol Sci. (2020) 21(4). doi: 10.3390/ijms21041178 32053970 PMC7072735

[89] Febbraio MA Pedersen BK . Muscle-derived interleukin-6: mechanisms for activation and possible biological roles. FASEB J. (2002) 16:1335–47. doi: 10.1096/fj.01-0876rev 12205025

[90] Pedersen BK Febbraio MA . Muscles, exercise and obesity: skeletal muscle as a secretory organ. Nat Rev Endocrinol. (2012) 8:457–65. doi: 10.1038/nrendo.2012.49 22473333

[91] Idorn M Hojman P . Exercise-dependent regulation of NK cells in cancer protection. Trends Mol Med. (2016) 22:565–77. doi: 10.1016/j.molmed.2016.05.007 27262760

[92] Pedersen L Idorn M Olofsson GH Lauenborg B Nookaew I Hansen RH . Voluntary running suppresses tumor growth through epinephrine- and IL-6-dependent NK cell mobilization and redistribution. Cell Metab. (2016) 23:554–62. doi: 10.1016/j.cmet.2016.01.011 26895752

[93] Fiuza-Luces C Valenzuela PL Gálvez BG Ramírez M López-Soto A Simpson RJ . The effect of physical exercise on anticancer immunity. Nat Rev Immunol. (2024) 24:282–93. doi: 10.1038/s41577-023-00943-0 37794239

[94] Frost RA Nystrom GJ Lang CH . Epinephrine stimulates IL-6 expression in skeletal muscle and C2C12 myoblasts: role of c-Jun NH2-terminal kinase and histone deacetylase activity. Am J Physiol Endocrinol Metab. (2004) 286:E809–17. doi: 10.1097/00024382-200406002-00239 14722032

[95] Kappel M Tvede N Galbo H Haahr PM Kjaer M Linstow M . Evidence that the effect of physical exercise on NK cell activity is mediated by epinephrine. J Appl Physiol (1985). (1991) 70:2530–4. doi: 10.1152/jappl.1991.70.6.2530 1885446

[96] Dimitrov S Lange T Born J . Selective mobilization of cytotoxic leukocytes by epinephrine. J Immunol. (2010) 184:503–11. doi: 10.4049/jimmunol.0902189 19949113

[97] Bigley AB Rezvani K Chew C Sekine T Pistillo M Crucian B . Acute exercise preferentially redeploys NK-cells with a highly-differentiated phenotype and augments cytotoxicity against lymphoma and multiple myeloma target cells. Brain Behav Immun. (2014) 39:160–71. doi: 10.1016/j.bbi.2013.10.030 24200514

[98] Qin B He Z Xie L Feng S Ye J Gui J . The augmentation of cytotoxic immune cell functionality through physical exertion bolsters the potency of chemotherapy in models of mammary carcinoma. Cancer Med. (2024) 13:e6951. doi: 10.1002/cam4.6951 38234174 PMC10905332

[99] Lakier Smith L . Overtraining, excessive exercise, and altered immunity: is this a T helper-1 versus T helper-2 lymphocyte response? Sports Med. (2003) 33:347–64. doi: 10.2165/00007256-200333050-00002 12696983

[100] Jin S Han S Wang N Yang M Chen C . Acute aerobic exercise alters serum protein distribution in colorectal cancer patients. Front Oncol. (2025) 15:1586344. doi: 10.3389/fonc.2025.1586344 40270615 PMC12014742

[101] Mao J Qiu X Zhang Y Wang C Yang X Li Q . A systematic review and meta-analysis of randomized controlled trials for physical activity among colorectal cancer survivors: directions for future research. PeerJ. (2025) 13:e18892. doi: 10.7717/peerj.18892 39902324 PMC11789654

[102] Machado P Morgado M Raposo J Mendes M Silva CG Morais N . Effectiveness of exercise training on cancer-related fatigue in colorectal cancer survivors: a systematic review and meta-analysis of randomized controlled trials. Support Care Cancer. (2022) 30:5601–13. doi: 10.1007/s00520-022-06856-3 35107601

[103] Ranjbar K Ballarò R Bover Q Pin F Beltrà M Penna F . Combined exercise training positively affects muscle wasting in tumor-bearing mice. Med Sci Sports Exerc. (2019) 51:1387–95. doi: 10.1249/mss.0000000000001916 30724848

[104] al-Majid S McCarthy DO . Resistance exercise training attenuates wasting of the extensor digitorum longus muscle in mice bearing the colon-26 adenocarcinoma. Biol Res Nurs. (2001) 2:155–66. doi: 10.1177/109980040100200301 11547537

[105] Pigna E Berardi E Aulino P Rizzuto E Zampieri S Carraro U . Aerobic exercise and pharmacological treatments counteract cachexia by modulating autophagy in colon cancer. Sci Rep. (2016) 6:26991. doi: 10.1038/srep26991 27244599 PMC4886631

[106] Tatebayashi D Himori K Yamada R Ashida Y Miyazaki M Yamada T . High-intensity eccentric training ameliorates muscle wasting in colon 26 tumor-bearing mice. PloS One. (2018) 13:e0199050. doi: 10.1371/journal.pone.0199050 29894511 PMC5997314

[107] Wood NR Garritson J Mathias A Haughian JM Hayward R . Moderate intensity endurance and resistance exercise attenuates cachexia in tumor-bearing mice. Anticancer Res. (2022) 42:397–405. doi: 10.21873/anticanres.15498 34969750

[108] Orange ST Jordan AR Odell A Kavanagh O Hicks KM Eaglen T . Acute aerobic exercise-conditioned serum reduces colon cancer cell proliferation *in vitro* through interleukin-6-induced regulation of DNA damage. Int J Cancer. (2022) 151:265–74. doi: 10.1002/ijc.33982 35213038 PMC9314683

[109] Brown JC Compton SLE Kang A Jayaraman A Gilmore LA Kirby BJ . Effects of exercise on inflammation, circulating tumor cells, and circulating tumor DNA in colorectal cancer. J Sport Health Sci. (2025) 14:101036. doi: 10.1016/j.jshs.2025.101036 40107449 PMC12341639

[110] Devin JL Hill MM Mourtzakis M Quadrilatero J Jenkins DG Skinner TL . Acute high intensity interval exercise reduces colon cancer cell growth. J Physiol. (2019) 597:2177–84. doi: 10.1113/jp277648 30812059 PMC6462486

[111] Landi F Marzetti E Martone AM Bernabei R Onder G . Exercise as a remedy for sarcopenia. Curr Opin Clin Nutr Metab Care. (2014) 17:25–31. doi: 10.1097/mco.0000000000000018 24310054

[112] Drake JC Wilson RJ Yan Z . Molecular mechanisms for mitochondrial adaptation to exercise training in skeletal muscle. FASEB J. (2016) 30:13–22. doi: 10.1096/fj.15-276337 26370848 PMC6137621

[113] Ruderman NB Keller C Richard AM Saha AK Luo Z Xiang X . Interleukin-6 regulation of AMP-activated protein kinase. Potential role in the systemic response to exercise and prevention of the metabolic syndrome. Diabetes. (2006) 55:S48–54. doi: 10.2337/db06-s007 17130644

[114] Nash D Hughes MG Butcher L Aicheler R Smith P Cullen T . IL-6 signaling in acute exercise and chronic training: Potential consequences for health and athletic performance. Scand J Med Sci Sports. (2023) 33:4–19. doi: 10.1057/9781137292155_8 36168944 PMC10092579

[115] Winder WW . Energy-sensing and signaling by AMP-activated protein kinase in skeletal muscle. J Appl Physiol (1985). (2001) 91:1017–28. doi: 10.1152/jappl.2001.91.3.1017 11509493

[116] McConell GK Lee-Young RS Chen ZP Stepto NK Huynh NN Stephens TJ . Short-term exercise training in humans reduces AMPK signalling during prolonged exercise independent of muscle glycogen. J Physiol. (2005) 568:665–76. doi: 10.1113/jphysiol.2005.089839 16051629 PMC1474728

[117] Garritson J Krynski L Haverbeck L Haughian JM Pullen NA Hayward R . Physical activity delays accumulation of immunosuppressive myeloid-derived suppressor cells. PloS One. (2020) 15:e0234548. doi: 10.1371/journal.pone.0234548 32542046 PMC7295224

[118] Pedersen BK Fischer CP . Beneficial health effects of exercise--the role of IL-6 as a myokine. Trends Pharmacol Sci. (2007) 28:152–6. doi: 10.1016/j.tips.2007.02.002 17331593

[119] Aderka D Le JM Vilcek J . IL-6 inhibits lipopolysaccharide-induced tumor necrosis factor production in cultured human monocytes, U937 cells, and in mice. J Immunol. (1989) 143:3517–23. doi: 10.4049/jimmunol.143.11.3517 2584704

[120] Starkie R Ostrowski SR Jauffred S Febbraio M Pedersen BK . Exercise and IL-6 infusion inhibit endotoxin-induced TNF-alpha production in humans. FASEB J. (2003) 17:884–6. doi: 10.1096/fj.02-0670fje 12626436

[121] Reihmane D Jurka A Tretjakovs P Dela F . Increase in IL-6, TNF-α, and MMP-9, but not sICAM-1, concentrations depends on exercise duration. Eur J Appl Physiol. (2013) 113:851–8. doi: 10.1007/s00421-012-2491-9 22990627

[122] Paolucci EM Loukov D Bowdish DME Heisz JJ . Exercise reduces depression and inflammation but intensity matters. Biol Psychol. (2018) 133:79–84. doi: 10.1016/j.biopsycho.2018.01.015 29408464

[123] Wei S Lv X Xu Y Ding S . Exercise-modulated IL-6 in cancer cachexia: molecular mechanisms and therapeutic potential. J Transl Med. (2026) 24:162. doi: 10.1186/s12967-026-07690-5 41527122 PMC12888731

[124] Thompson DK Huffman KM Kraus WE Kraus VB . Critical appraisal of four IL-6 immunoassays. PloS One. (2012) 7:e30659. doi: 10.1371/journal.pone.0030659 22347395 PMC3276568

[125] Lyu Y Han T Zhang Z Wu Y Guan Q Hong E . Procalcitonin and interleukin- 6 in predicting prognosis of sepsis patients with cancer. Support Care Cancer. (2025) 33:404. doi: 10.1007/s00520-025-09464-z 40261444

[126] Martin H Olander B Norman M . Reactive hyperemia and interleukin 6, interleukin 8, and tumor necrosis factor-alpha in the diagnosis of early-onset neonatal sepsis. Pediatrics. (2001) 108:E61. doi: 10.1542/peds.108.4.e61 11581469

[127] Brown JC Compton SLE Meyerhardt JA Spielmann G Yang S . The dose-response effect of aerobic exercise on inflammation in colon cancer survivors. Front Oncol. (2023) 13:1257767. doi: 10.3389/fonc.2023.1257767 38148846 PMC10750999

